# Transcriptome of Russet Norkotah and its clonal selection, TXNS278

**DOI:** 10.1186/s13104-018-3254-4

**Published:** 2018-03-01

**Authors:** Julien Levy, Cecilia Tamborindeguy, Giridhar Athrey, Douglas C. Scheuring, Jeffrey W. Koym, J. Creighton Miller

**Affiliations:** 1 Department of Horticultural Sciences, Texas A&M University, College Station, TX USA; 2 Department of Entomology, Texas A&M University, College Station, TX USA; 3 Department of Poultry Sciences, Texas A&M University, College Station, TX USA; 4 Department of Horticultural Sciences, Texas A&M AgriLife Research, Lubbock, TX USA

**Keywords:** Russet Norkotah, Clonal selection, RNA-seq, Genomic

## Abstract

**Objectives:**

Potato has a large genetic diversity. This diversity is in part due to somaclonal variability that appears within potato selections for which tubers are used as seeds. However, the potato tetraploid genome, as well as the use of tubers for crop propagation, does not allow for easy genetic studies. The objective is to gain knowledge at the genomic level from standard Russet Norkotah and a subclonal Russet Norkotah selection TXNS278.

**Data description:**

In this report, we used RNA-seq, which allows genome-wide gene expression analysis to sequence the transcriptomes of the subclonal Russet Norkotah selection TXNS278 with standard Russet Norkotah grown in commercial fields. Among the selections, TXNS278 appeared in a multi-year analysis in Texas as a top No 1 yielding variety. Russet Norkotah and TXNS278 leaf and root transcriptomes were sequenced at two time points during growing season.

**Electronic supplementary material:**

The online version of this article (10.1186/s13104-018-3254-4) contains supplementary material, which is available to authorized users.

## Objective

Potato (*Solanum tuberosum* L.) is one of the most important human food crops, and its production is increasing [1]. Potato genetic diversity is partially the consequence of a tetraploid genome, as well as propagation using the potato tubers and not true seeds. This allows the development of new mutations in cultivated populations called somaclonal variations [2]. The cultivar Russet Norkotah (RN) is the 2nd most popular russet in the US [3]. In 1989, the Texas Potato Breeding and Variety Development Program made strain selections to improve the Russet Norkotah cultivar [4]. Strains were first selected for criteria such as large vines (giant hill). After these initial selections, the remainder of the selection cycles were based on tuber type and yield across multiple locations in Texas. By 1998, six clones remained in the program, including the Texas Russet Norkotah Strain 278 (TXNS278).

RN and TXNS278 were grown from 1989 to 2007 for 18 generations in 15 locations in Texas. It was determined that TXNS278 is genetically uniform and stable from generation to generation with no evidence of variants [5, 6].

The objective of this study was to compare differentially expressed genes between RN and TXNS278. Genomic differences resulting from somaclonal selection of potato are difficult to characterize. For instance, to date, no genetic differences have been identified between RN or any other Texas selections including TXNS278 using isozymes, RFLP, and AFLP markers [6].

In this dataset, next-generation sequencing was used to sequence root and leaf transcriptomes of the two potato cultivars at two time points in the growing season. RNAseq is a powerful tool for research, which facilitates identifying differences in gene expression between cultivars in order to gain insight into their genomic and physiological differences.

## Data description

### Plant material for transcriptome analysis

Russet Norkotah and the clonal selection TXNS278 were grown near Springlake, TX as part of the Texas Potato Breeding and Variety Development Program in the summer of 2013. The seeds of Russet Norkotah were obtained from Oregon State University—Klamath Basin Research & Extension Center. The seed of TXNS278 was obtained from Dr. David Holm, Colorado State University at the San Luis Valley Research Center in Colorado. The plots were planted April 1, 2013 from tuber seed obtained from certified seed growers. Tissues were sampled on July 6th (T1, 66 days after planting) and on July 24th (T2, 84 days after planting). Tissues were stored in a tube in RNA*later* Stabilization Solution (Thermo Fischer Scientific, Waltham, MA) and placed on ice in a cooler before transportation to the laboratory, where tissues were frozen upon arrival and stored at − 20 °C. Leaves and roots from different plants within the same plot were sampled independently at both time points. Plots of TXNS278 and RN were separated by two feet.

### RNA extraction

RNA was isolated from individual samples using the Qiagen plant RNeasy kit. DNAse treatment was performed according to manufacturer recommendations (Qiagen, Hilden, Germany). RNA was quantified using an Infinite 200 PRO NanoQuant (Tecan, Mannedorf, Switzerland), and quality was verified by Bioanalyzer (Texas A&M AgriLife Genomics & Bioinformatics Service, College Station, TX).

### RNA sequencing

A total of 24 RNA samples were submitted to the AgriLife Genomics & Bioinformatics Service. After RNA quality evaluation, three independent RNA samples (biological replicates) were pooled. Poly(A) RNA enrichment, library construction, and RNA sequencing from each pool were performed at the AgriLife Genomics & Bioinformatics Service.

One library was made for each potato cultivar (RN and TXNS278) at each time point (T1 and T2) and from each tissue (leaf and root). Therefore, a total of eight libraries were made using the TruSeq Kit (Illumina, San Diego, CA). The sequencing was performed using 100 single-end reads on one lane of the Illumina Hiseq-2000 platform.

The libraries were made publicly available through NCBI and can be found at the following address https://www.ncbi.nlm.nih.gov/geo/query/acc.cgi?&acc=GSE87857. A summary of the sequencing results is described in Table 1.

**Table 1:** Overview of data files

Label	Name of data file/data set	File types (file extension)	Data repository and identifier (DOI or accession number)	License
Data file 1	GSM2341973 RN-Leaf-T1	GTF (.gtf.gz)	NCBI GEO (GSM2341973)	N/A
Data file 2	GSM2341974 TXNS278-Leaf-T1	GTF (.gtf.gz)	NCBI GEO (GSM2341974)	N/A
Data file 3	GSM2341975 TXNS278-Leaf-T2	GTF (.gtf.gz)	NCBI GEO (GSM2341975)	N/A
Data file 4	GSM2341976 RN-Root-T1	GTF (.gtf.gz)	NCBI GEO (GSM2341976)	N/A
Data file 5	GSM2341977 TXNS278-Root-T1	GTF (.gtf.gz)	NCBI GEO (GSM2341977)	N/A
Data file 6	GSM2341978 RN-Root-T2	GTF (.gtf.gz)	NCBI GEO (GSM2341978)	N/A
Data file 7	GSM2341979 TXNS278-Root-T2	GTF (.gtf.gz)	NCBI GEO (GSM2341979)	N/A
Data file 8	GSM2982237 RN-Leaf-T2	GTF (.gtf.gz)	NCBI GEO (GSM2982237)	N/A

### Mapping of RN and TXNS278 transcriptomes to the potato genome

Leaf and root samples were collected from a commercial field near Springlake, TX in 2013. Two time points were tested, based on plant development: T1 (full flowering) and T2 (senescing plants).

Over 176 million reads that passed the quality filters were obtained (Additional file 1: Table S1), with an average of 22 million reads per library. The reads were mapped to the *S. tuberosum* double haploid DMI3.4 genome ensembl19 using Tophat2 in CyVerse (iPlantcollaborative.org). Only 33% of the reads from the library RN-LeafT2 mapped to the potato genome (Table 1 data file 8). After exclusion of this library, a minimum of 66.3% of reads mapped to the potato genome, from which 55 to 68% were uniquely aligned reads (Additional file 1: Table S1). The samples used for the data file 8 were infected with potato virus and only 33% of the reads matched the potato genome. Consequently these data should be excluded from further analyses. Interestingly, a higher percentage of unique mapped reads were obtained from the root libraries (62–67%). This difference might be related to higher rRNA levels in leaves than in roots in spite of the mRNA enrichment.

### Limitation

The libraries were sequenced on pooled samples from three biological replicates; this reduced the cost of sequencing multiple samples, but limited the statistical power of the analysis. Nevertheless, several studies have been published using pooled samples, and different software has been designed to analyze pooled samples. This sequencing provides valuable RNAseq data of Russet Norkotah genes for future sequencing of cultivated potato species and annotation of genes and transcripts.

## Additional file

**Additional file 1: Table S1.** Overview of the mapping results.

## Abbreviations

RN: Russet Norkotah
TXNS278: Texas Russet Norkotah Strain 278
